# Platelet-Rich Plasma Lysate-Incorporating Gelatin Hydrogel as a Scaffold for Bone Reconstruction

**DOI:** 10.3390/bioengineering9100513

**Published:** 2022-09-29

**Authors:** Meral Nadra, Wanting Niu, Motoichi Kurisawa, Dominique Rousson, Myron Spector

**Affiliations:** 1 Department of Restorative Dentistry and Biomaterials Sciences, Harvard School of Dental Medicine, Boston, MA 02115, USA; 2 Tissue Engineering/Regenerative Medicine, VA Boston Healthcare System, Boston, MA 02130, USA; 3 Orthopedic Surgery, Brigham and Women’s Hospital, Harvard Medical School, Boston, MA 02115, USA; 4 Japan Advanced Institute of Science and Technology, Nomi 923-1292, Japan

**Keywords:** biomaterial, gelatin, bone regeneration, hydrogel, growth factors, tissue engineering, bone substitute, sustainable release, platelet-rich plasma lysate, regenerative medicine

## Abstract

In implant dentistry, large vertical and horizontal alveolar ridge deficiencies in mandibular and maxillary bone are challenges that clinicians continue to face. One of the limitations of porous blocks for reconstruction of bone in large defects in the oral cavity, and in the musculoskeletal system, is that fibrin clot does not adequately fill the interior pores and does not persist long enough to accommodate cell migration into the center of the block. The objective of our work was to develop a gelatin-based gel incorporating platelet-rich plasma (PRP) lysate, to mimic the role that a blood clot would normally play to attract and accommodate the migration of host osteoprogenitor and endothelial cells into the scaffold, thereby facilitating bone reconstruction. A conjugate of gelatin (Gtn) and hydroxyphenyl propionic acid (HPA), an amino-acid-like molecule, was commended for this application because of its ability to undergo enzyme-mediated covalent cross-linking to form a hydrogel in vivo, after being injected as a liquid. The initiation and propagation of cross-linking were under the control of horseradish peroxidase and hydrogen peroxide, respectively. The objectives of this in vitro study were directed toward evaluating: (1) the migration of rat mesenchymal stem cells (MSCs) into Gtn–HPA gel under the influence of rat PRP lysate or recombinant platelet-derived growth factor (PDGF)-BB incorporated into the gel; (2) the differentiation of MSCs, incorporated into the gel, into osteogenic cells under the influence of PRP lysate and PDGF-BB; and (3) the release kinetics of PDGF-BB from gels incorporating two formulations of PRP lysate and recombinant PDGF-BB. **Results**: The number of MSCs migrating into the hydrogel was significantly (3-fold) higher in the hydrogel group incorporating PRP lysate compared to the PDGF-BB and the blank gel control groups. For the differentiation/osteogenesis assay, the osteocalcin-positive cell area percentage was significantly higher in both the gel/PRP and gel/PDGF-BB groups, compared to the two control groups: cells in the blank gels grown in cell expansion medium and in osteogenic medium. Results of the ELISA release assay indicated that Gtn–HPA acted as an effective delivery vehicle for the sustained release of PDGF-BB from two different PRP lysate batches, with about 60% of the original PDGF-BB amount in the two groups remaining in the gel at 28 days. **Conclusions**: Gtn–HPA accommodates MSC migration. PRP-lysate-incorporating hydrogels chemoattract increased MSC migration into the Gtn–HPA compared to the blank gel. PRP-lysate- and the PDGF-BB-incorporating gels stimulate osteogenic differentiation of the MSCs. The release of the growth factors from Gtn–HPA containing PRP lysate can extend over the period of time (weeks) necessary for bone reconstruction. The findings demonstrate that Gtn–HPA can serve as both a scaffold for cell migration and a delivery vehicle that allows sustained and controlled release of the incorporated therapeutic agent over extended periods of time. These findings commend Gtn–HPA incorporating PRP lysate for infusion into porous calcium phosphate blocks for vertical and horizontal ridge reconstruction, and for other musculoskeletal applications.

## 1. Introduction

Dental implant therapy has proven to be of great value for treating partially and fully edentulous patients. However, following extraction of teeth, alveolar ridge remodeling usually results in vertical and/or horizontal bone loss. Adequate alveolar bone volume and adequate soft tissue are needed for proper implant placement ensuring functional and esthetic long-term outcomes [1,2,3,4]. Inadequate volume of alveolar bone to support implants, post-extraction, can be exacerbated by previous trauma, dental infections, developmental or congenital malformations, and periodontal disease. 

There are a wide array of materials and techniques that are currently being used in the clinic for alveolar ridge augmentation. A recent systematic review concluded that although vertical ridge augmentation is a feasible treatment for managing alveolar ridge deficiencies, complications are common [5]. The complex anatomical features and the poor blood supply, especially in posterior atrophic mandibles, can jeopardize the predictability and outcomes of the available ridge augmentation techniques [6]. Many such studies have highlighted the need for tissue engineering/regenerative medicine advances in the field of bone regeneration including the use of porous biomaterials incorporating biologics [7,8,9].

One such biologic, platelet-rich plasma (PRP) lysate (also referred to as platelet lysate), is proving to be a promising preparation for bone regeneration [10]. PRP and PRP products are natural, autologous sources of growth factors that are known to stimulate cell processes related to bone regeneration and angiogenesis. PRP lysate has attracted attention because it is an acellular preparation containing the growth factors carried by platelets but does not include the lipid/membranous material of the platelets. A recent review has summarized the clinical applications for which platelet lysate is being investigated and the methods of its preparation [11]. A study of one such application concluded that among the different PRP formulations used in the study, the PRP lysate formulation had higher antimicrobial effect against synovial fluid biofilm [12]. Another in vitro study of the impact of PRP lysate on cartilage regeneration showed that platelet lysate preparations proved to have a beneficial therapeutic impact on chondrogenesis when used as an adjunct treatment with autologous chondrocyte implantation (ACI) [13]. That study recommended the use of PRP lysate to synergize the effect of PRP in reducing inflammation and promoting chondrogenesis [13,14]. A variety of methods have been employed for the preparation of PRP and PRP lysate for bone regeneration [10,15]; our study used a standardized protocol for rat PRP lysate developed by our lab (described below). 

While recent work has shown that combinations of growth factors loaded in a porous calcium phosphate (CaP) scaffold can promote bone regeneration [16], further research is needed to determine the optimal combination of scaffold and biologic for alveolar bone reconstruction [17]. New strategies are needed to enhance the inadequate efficacy of bone formation in porous blocks employed to treat alveolar defects [18]. These limitations of porous blocks for bone reconstruction might, in part, be due to the inability of a fibrin clot to fill the pores of the block. One approach to deal with this issue may be to infuse the pores with a hydrogel to replace the role of a fibrin clot.

A wide array of hydrogels has been developed for various applications including delivery of drugs and biologics and replacement of soft tissues [9,19,20,21]. These in situ gellable hydrogels, which are injected in a liquid form to undergo gelation in vivo, offer particular advantages: (1) they allow for homogenous incorporation of therapeutic molecules or cells; (2) they can be delivered to the defect site through a small bore needle; and (3) they can conform to irregular defect shapes. Enzyme-mediated covalently cross-linked gelatin gels have the advantages of providing adhesion ligands for a wide array of cell types and of undergoing proteolytic degradation. The specific gelatin conjugate that we used undergoes covalent cross-linking in vivo initiated by horseradish peroxide (HRP) and propagated by peroxide (H_2_O_2_), thus enabling tunable gelation rate, mechanical properties, and degradation rate [21,22,23,24]. 

As presented, one of the unmet challenges inherent in using porous scaffolds for the reconstruction of bone in relatively large volume defects is the lack of a suitable matrix that ensures sufficient attraction and accommodation of the migration of host osteoprogenitor and endothelial cells into the interior structure of the implanted scaffold. During normal physiologic wound healing, it is the blood clot that initially forms in a surgical defect in bone that provides (1) a fibrin matrix to which host cells can adhere and on which they can migrate, and (2) platelets that release growth factors, such as platelet-derived growth factor (PDGF-BB), which attracts such cells into the defect and stimulates their proliferation and differentiation. These two critically important roles of the blood clot in defects implanted with porous scaffolds are limited by (1) insufficient bleeding into the interior of the scaffold to form a robust clot and (2) fibrinolysis of the clot that occurs before the host cells have been able to migrate into the interior of the block. With this motivation of implementing a substitute for the fibrin clot, which could be infused into a porous scaffold prior to its implantation, we developed a gelatin-based gel incorporating platelet-rich plasma lysate.

There were three objectives for this in vitro study. The first was to determine if Gtn–HPA can accommodate the migration of rat mesenchymal stem cells (MSCs), under the influence of a chemoattractant incorporated into the gel. Associated with this objective was our hypothesis that PRP lysate could serve as this chemoattractant for MSCs. This objective was achieved by evaluating the number of MSCs migrating into Gtn–HPA gel under the influence of rat PRP lysate incorporated into the gel. Gels incorporating recombinant platelet-derived growth factor (PDGF)-BB and without growth factor incorporation (blank gels) served as positive and negative control groups, respectively. The second objective was to determine if MSCs contained within the gel retained their ability to undergo osteogenic differentiation. Associated with this objective was our hypothesis that this osteogenic differentiation could be stimulated by growth factors incorporated into the gel, including recombinant PDGF-BB and those in PRP lysate. The third objective was to determine if the Gtn–HPA gel can serve as an effective carrier for the prolonged release of growth factors such as PDGF-BB for more than 4 weeks.

## 2. Materials and Methods

### 2.1. Rat MSC Expansion and Differentiation Assay

Rat MSCs used in this study were isolated in our lab from tibia and femurs of adult Sprague Dawley (SD) rats > 3 months old following protocol [25]. The isolated cells were cultured in expansion medium: Dulbecco’s Modified Eagle Medium-low glucose (DMEM-LG; Gibco by Life technologies, Carlsbad, CA, USA) supplemented with 10% fetal bovine serum (FBS, VWR, Waltham, MA, USA), 10 ng/mL recombinant murine fibroblast growth factor (FGF)-2 (PeproTech, East Windsor, NJ, USA), 100 U/mL penicillin, and 100 µg/mL streptomycin (Millipore-Sigma, Waltham, MA, USA). The cells were used at passages 3 or 4 (P3/P4) for all experiments. Adipogenic and osteogenic differentiations were performed beforehand to confirm the stemness of the isolated cells. Cultured cells at passage 3 were seeded in a six-well cell culture plate. When the cells reached 80% confluence, the expansion medium was replaced by osteogenic differentiation medium or adipogenic differentiation medium, respectively. For the osteogenic differentiation medium: DMEM-LG supplemented with 10% FBS, 0.05 mM ascorbic acid 10 mM ß-glycerol phosphate, and 100 nM dexamethasone [26]. Adipogenic differentiation medium: DMEM-high glucose (HG) supplemented with 10% FBS, 1 µM dexamethasone, 10 µg/mL insulin, 500 µM 3-isobutyl-1-methylxathine (IBMX, Millipore-Sigma, Waltham, MA, USA), and 1 µM indomethacin (Millipore-Sigma, Waltham, MA, USA). The medium was changed every other day for 21 days. After 21 days, cells were fixed using 4% paraformaldehyde (PFA, Thermo Fisher, Waltham, MA, USA) overnight. Histochemical staining was then performed to assess differentiation based on morphology.

### 2.2. PRP Lysate Preparation

**Figure 1:**
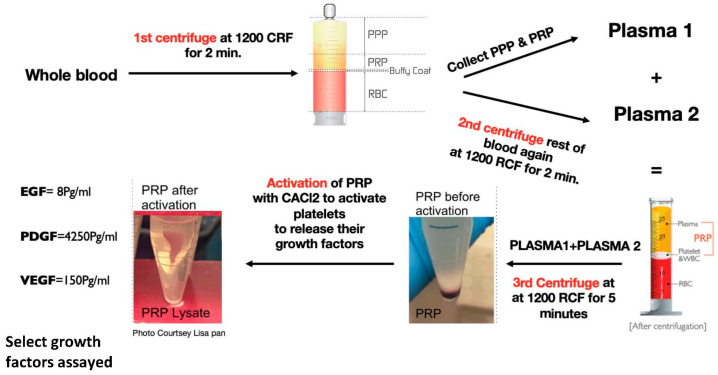
Diagram showing the processing method performed in our lab to obtain PRP Lysate. ELISA quantified growth factors in the PRP lysate used in the migration assay are shown on the left.

PRP from blood extracted from 15 SD rats (9 males and 6 females) > 3 months old was extracted and prepared using a standardized repeatable protocol especially developed for rat blood by our lab (Figure 1). Whole rat blood (8 mL) was collected into sterile syringes containing 2 mL of anticoagulant citrate dextrose solution (ACD to whole blood ratio of 1:4), the whole blood was initially centrifuged at 1200 RCF for 2 min. Platelet-poor plasma (PPP) and PRP layers were collected in a separate tube and labelled “plasma 1”, as shown in Figure 1. When second centrifugation for the remaining blood at 1200 RCF was performed, PPP and PRP layers were collected and labelled “plasma 2”. Plasma 1 and plasma 2 were then added together in another tube for the 3rd centrifugation at 1200 RCF for 5 min. The PRP prepared from 15 rats was pooled together to reduce differences among individuals. Blood cell counting was performed on a hematology analyzer (Sysmex, XP-300, Lincolnshire, NE, USA) for whole blood and PRP. Platelets were activated by addition of 250 µL of 10% CaCl_2_ into 10 mL of PRP, and incubation at 37 °C for 1 h for activation according to the platelet activation protocol by Textor et al., 2012 [27]. The formed fibrin clot was removed, and the PRP lysate was frozen at −20 °C for future use. 

### 2.3. PRP Standardization and Growth Factors Quantification

An aliquot of the PRP lysates prepared was taken for enzyme-linked immunosorbent assay (ELISA) testing to quantify the concentration of PDGF-BB present for each experiment. The assay was performed using a mouse/rat (PDGF-BB) ELISA kit (R&D Systems, Minneapolis, MN, USA) following the manufacture’s protocol (Rat-PDGF Kit R&D USA). The wells of the plates were diluted with the capture antibody, incubated, and washed. Samples in reagent diluent were then added to each well, washed to add detection antibody, and then washed again to add the streptavidin-HRP for 20 min covered from light. After that, they were washed, and 100 µL of substrate solution was added to each well for another 20-min incubation period. Finally, the stop solution was added, plates were tapped gently per the manufacturer’s instructions, and the optical density of each well was immediately read by a microplate reader (SpectraMax, ix3, Molecular Device, San Jose, CA, USA).

### 2.4. Gtn–HPA Gel Formation

Wang et al. developed Gtn–HPA hydrogels to be used as scaffolds for tissue engineering applications; characterization of the gel and the role of its tunable stiffness for osteogenic differentiation were studied by the same group in 2012. Gtn–HPA [22,24,28,29] was obtained from Dr. Motoichi Kurisawa, currently at the Japan Advanced Institute of Science and Technology, Nomi, Japan. Lyophilized gelatin conjugate (Gtn) and 3-(4-hydroxyphenyl) propionic acid (HPA) were initially prepared from raw materials. Gelation of Gtn–HPA hydrogel was the result of covalently cross-linking the Gtn–HPA conjugate with horseradish peroxidase (HRP) and hydrogen peroxide (H_2_O_2_) through the HRP-catalyzed crosslinking mechanism. 

Gtn–HPA hydrogels were produced by first preparing a Gtn–HPA conjugate solution for the intended final gelatin concentration planned for the migration assay experiment (2%). 

In this study, 4% Gtn–HPA solution was prepared first by dissolving the lyophilized Gtn–HPA conjugate in phosphate-buffered saline (PBS) at 37 °C, and then mixing with cell suspension or DMEM-LG (Life technologies, USA) to reach a final concentration of 2%. The gel formulation used was crosslinked by 0.1 U/mL HRP (Wako Chemicals, USA) and 1.2 mM H_2_O_2_ (Sigma-Aldrich, USA). Our prior work demonstrated that cells remain viable through the H_2_O_2_-propagated covalent cross-linking of Gtn–HPA gels. The viability of neural stem cells (NSCs) was evaluated in Gtn–HPA gels exposed to 0.85, 1.0, 1.2, and 1.7 mM H_2_O_2_ during the covalent cross-linking (gelation) process [30]. The viability of gel-encapsulated NSCs remained as high as the control cultures in conventional monolayer culture without exposure to H_2_O_2_—~95–97% cell viability for monolayer cultures—in gels cross-linked with 0.85, 1.0, and 1.2 mM H_2_O_2_. Only gels containing 1.7 mM H_2_O_2_ displayed a slight decrease in cell viability to ~93% [30]. In a recent study employing caprine bone-marrow-derived MSCs, 99% of the cells exposed to the Gtn–HPA cross-linking process using 1.2 mM H_2_O_2_ were found to remain viable using a live/dead cell assay [31].

## 3. Experimental Protocols

### 3.1. Core-Ring 3D Assembly Migration Assay (n = 6) [32]

After 4% Gtn–HPA was prepared as described earlier, the final 2% Gtn–HPA used for the experiment was obtained by adding pure DMEM (Life Technologies, USA) with a ratio of 1:1. A total 10% (*v*/*v*) PRP lysate was incorporated into the gels (less medium) for the PRP lysate group; as for the PDGF experimental group, 50 ng/mL of rat-PDGF-BB was incorporated into the hydrogel. Afterward, 250 µL of the 2% hydrogel was cast into syringe-cut molds as the core gel and left for gelation at 37 °C for 30 min. Then, MSC-seeded collagen gel rings were cast around each core gel. A total 350 µL of 0.8 mg/mL soluble rat tail type I collagen solution (BD, USA) incorporating the MSCs at a cell density of 0.2 million cells/mL was cast around each core Gtn–HPA gel and incubated for two hours at 37 °C. Finally, 1 mL of DMEM +10% FBS+ 100 U/mL penicillin and 100 µg/mL streptomycin (expansion medium) was added to the culture dishes and changed every other day.

After 10 days, each sample was fixed using 1 mL of 4% PFA at 4 °C overnight. Samples were then stained according to the manufacturer’s immunostaining protocol with 2-(4-Amidinophenyl)-6-indolecarbamidine dihydrochloride (DAPI) for nucleic acid staining diluted 1/200, anti α-smooth muscle actin (α-SMA) 5 µg/mL (Millipore-Sigma Aldrich catalog #A5228), anti-CD105 monoclonal antibody (Abcam, clone #0TI8A1) 1/1000 diluted, and antibody and Ki67 staining with 1/200 diluted (Abcam, clone# SP6). Alexa 488 or Cy3 labeled anti-mouse or rabbit antibodies were used as the secondary antibodies (Jackson ImmunoResearch, West Grove, USA). The time allowed for the primary and secondary antibodies to penetrate into the 3D casted hydrogels was 24 h. 

DAPI was used to counter stain the nuclei in order to facilitate the quantification of migrating MSCs; confirmation was provided by CD105 and α-SMA. Confocal laser scanning microscope (Nikon Eclipse C2+ laser scanning system, Japan) was used to image cells migrating into the 3D core gel across the interface between the collagen gel ring seeded with the MSCs and the core of Gtn–HPA. Images were then exported to Image J (Version IJ 2.0.0-rc, Fiji Java 1.8.0_172) software for quantification. First, the pixel/micron ratio shown on the microscope was used to set the scale using the analyze tool on image J for the analysis. After that, the image type selected was 8-bit and the adjust threshold tool was employed. The area where the line of demarcation was initially defined by the confocal microcopy was then selected and we used the analyze particles tool to quantify the DAPI-stained nuclei into the core gel. Finally, the resulted counts were then used for the statistical analysis.

### 3.2. Assessing Cell Osteogenic Differentiation in PRP-Lysate-Infused Cell-Seeded Hydrogel

Following are the experimental groups that were divided into 4 groups with N = 6, as follows, according to medium type and growth factors incorporated in the gel:

Group 1: “Control group”, in which an MSC-seeded Gtn–HPA hydrogel was cultured Cell expansion medium (CEM). 

Group 2: “Positive control”, in which an MSCs-seeded Gtn–HPA hydrogel was cultured in osteogenic medium (OM).

Group 3: PRP lysate was incorporated into MSCs-seeded Gtn–HPA hydrogel (10% volume) + OM.

Group 4: Recombinant rat-PDGF-BB was incorporated into MSCs-seeded Gtn–HPA hydrogel (50 ng/mL) and cultured in OM.

In each well of a 48-well plate, 200 µL of cell-seeded 2% Gtn–HPA cell-seeded hydrogels (with cell density of 10^5^ cells/mL) was cast and then incubated for 20 min in a cell incubator to complete gelation before adding medium.

After 28 days, all samples were fixed using 4% paraformaldehyde overnight. Immunostaining then was performed for 2 osteogenic differentiation markers: anti-osteocalcin antibody (Abcam, clone #: OC4-30) with the recommended manufacturer dilution 1/80, anti-SP7; Osterix antibody (Abcam, clone #: EPR21034) with dilution 1/500. In both cases, nuclei were counter stained by DAPI. The rest of the immunofluorescent staining protocol was the same as mentioned above in experiment I. For imaging, the same confocal microscopy mentioned above was used using the 10× macro-objective lens. This method has been tested previously by our lab and published by Niu et al., 2021 [31]. 

Images were exported to Image J for quantification. Area percentage covered by fluorescent signal of the selected final stage osteogenic marker, osteocalcin, in the hydrogel for each sample across the groups was calculated. The calculated percent area data were then used for the statistical analysis and comparison among the groups.

### 3.3. The Release Profile of PDGF-BB from Gels Incorporating PRP Lysate and Recombinant PDGF-BB

Studying the release of PDGF was important to determine whether the Gtn–HPA acts as a delivery vehicle that allows sustained and controlled release of the infused therapeutic agents over extended periods of time.

Four experimental groups were studied with N = 5 for each group:

(1) 2% Gtn–HPA blank hydrogel;
(2) PRP lysate preparation I incorporated into Gtn–HPA;
(3) PRP lysate preparation II incorporated into Gtn–HPA;
(4) Recombinant rat PDGF-BB (50 ng/mL) incorporated into Gtn–HPA.

PDGF-BB concentrations were quantified or the PRP lysate preparations I and II, 1561 pg/mL and 1604.1 pg/mL, respectively. 

## 4. Results

### 4.1. Cell Migration into the Gtn–HPA Hydrogel

The number of cells migrating into the gels containing PDGF-BB was ~25% higher than the number of cells found migrating into the blank control gels, demonstrating the chemoattractive influence of this growth factor on MSC migration. A notable finding was that the number of MSCs migrating into the hydrogel that incorporated PRP lysate was more than 200% higher than the number of cells migrating into the gels containing PDGF-BB, showing the beneficial effect of other growth factors contained in the PRP lysate on MSC migration.

**Figure 2:**
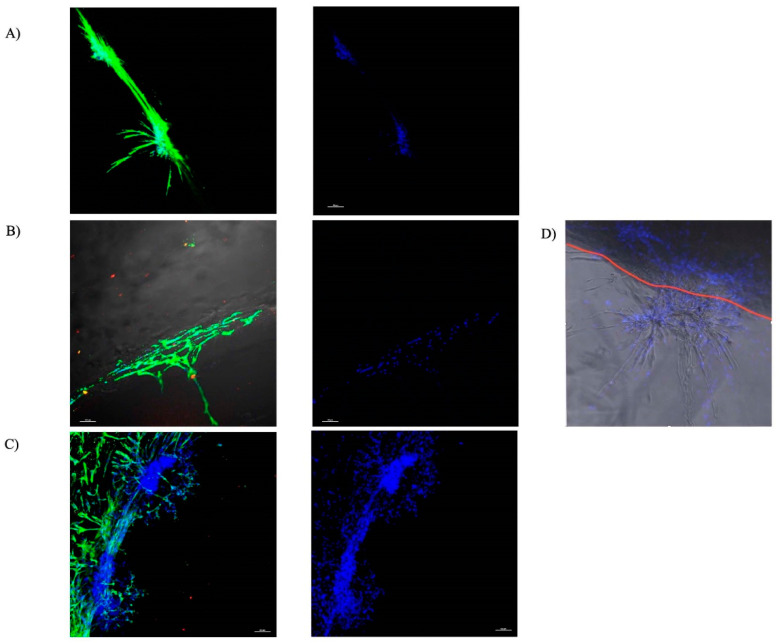
Representative images of the ring/core interface with rat MSCs expressing α-SMA migrating into the core Gtn–HPA. DAPI, blue; Anti α-SMA, green (Scale bar 100 μ). (**A**) Control Gtn–HPA gel. (**B**) Gtn–HPA incorporating PDGF-BB. (**C**) Gtn–HPA incorporating PRP lysate. (**D**) representative image illustrating the superimposed image from the confocal microscopy of one of the samples showing the chain migration and the branching of the chains as the cells migrate into the gel. The red line illustrates the demarcation of the interface between the collagen ring and the core-Gtn–HPA hydrogel.

As frequently reported in the literature, the MSCs reliably stained for α-SMA [33,34]. Particular migration patterns for all the groups (Figure 2) were evident, as depicted by the α-SMA staining of the cytoskeleton of the cells (Figure 2A). MSCs in the culture of the blank control gels (Figure 2A) and gels incorporating PDGF-BB (Figure 2B) were generally found along the surface of the gels, with few cells infiltrating the gel. 

There was a notable difference in the migration of MSCs into the gels incorporating PRP lysate group (Figure 2C). This migration pattern, reflective of chain migration, took the form of trees branching out across the ring–core interface in tuft-like structures towards the Gtn–HPA core gel. 

**Figure 3:**
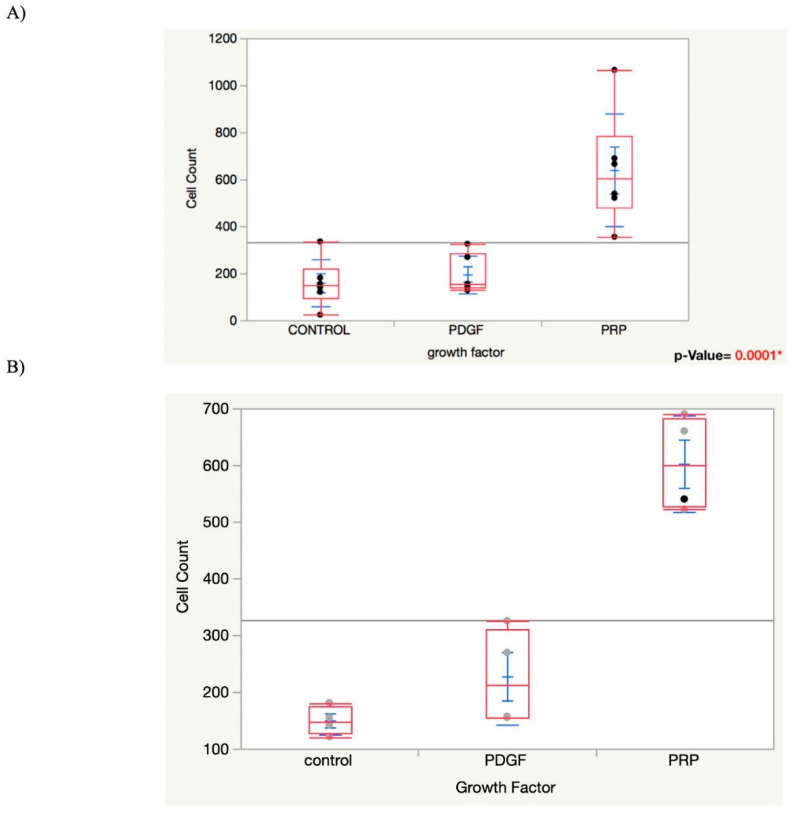
(**A**) Graph showing one-way ANOVA analysis for the migration assay “Cell Count” by “Growth Factor” among the groups. (**B**) Graph showing one-way ANOVA analysis for migration assay after removing the outliers. *p*-value < 0.0001*.

The statistical results of the migration assay showed that the number of MSCs migrating into the hydrogel was significantly higher in the gels that incorporated PRP lysate (640 ± 240) compared with the gels containing PDGF-BB (196 ± 81) and the blank gel controls (160 ± 41); n = 6. One-way ANOVA showed a significantly higher difference for the PRP lysate group than the control and PDGF groups with *p*-values of 0.0006 and 0.0003, respectively (Figure 3).

### 4.2. Osteogenic Differentiation of MSCs in the Gtn–HPA Hydrogel

**Figure 4:**
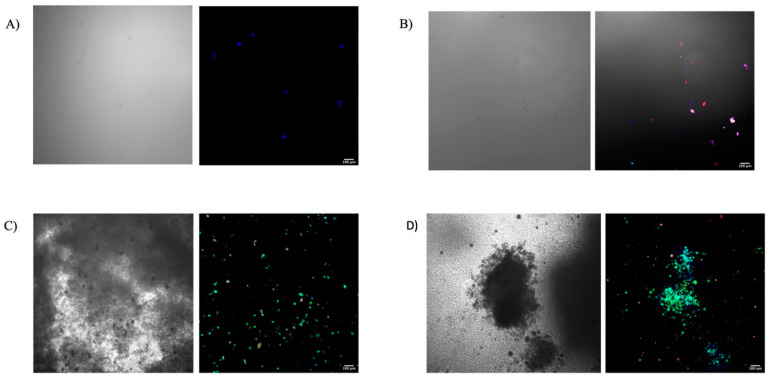
Representative DAPI (Blue)/Anti-Osteocalcin antibody (Green)/Anti-Osterix antibody (Red) immunostained cells in hydrogel samples for differentiation assay, showing the expression of Osteocalcin positive cells among the groups on the right side and the corresponding bright field images on the left side. (**A**) Control with CEM, (**B**) Control group with OM, (**C**) PDGF-BB group, (**D**) PRP lysate group.

Although the osteogenic medium groups showed differentiated osteogenic cells, the Osterix-positive cells were more evident than osteocalcin^+^ cells, as shown in (Figure 4). While there were no such Osterix cells in the blank gel groups, many such cells were found in the gels incorporating PDGF-BB and PRP lysate.

**Figure 5:**
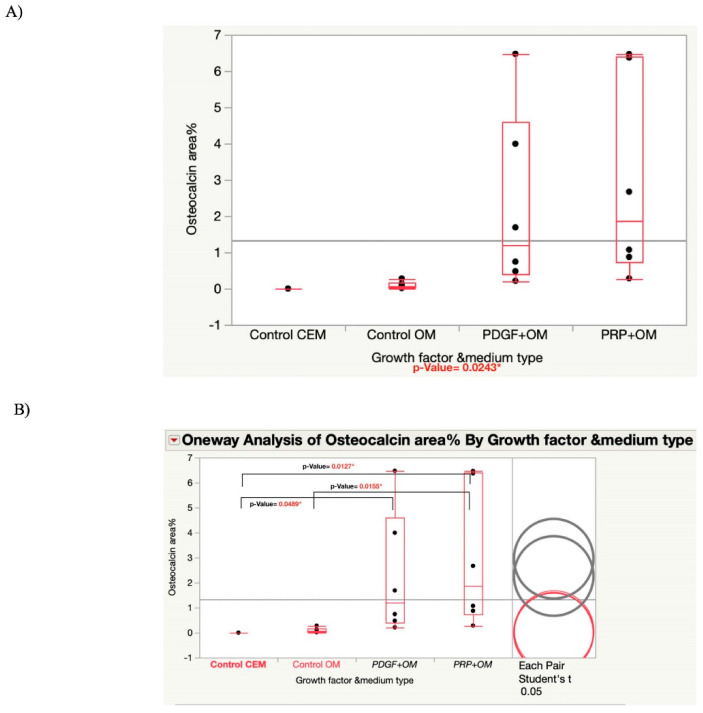
(**A**) Graph showing one-way ANOVA analysis of Osteocalcin fluorescence area% among the groups. (**B**) Graph showing one-way analysis of Osteocalcin fluorescence area% among the groups using pairwise student *t*-test. Significant differences are shown between the two groups using growth factors compared with the two control groups.

The quantitative statistical analysis of the area percentage covered by the osteocalcin positive MSCs seeded in the hydrogels was significantly higher in the growth-factors-infused hydrogels compared with the control groups where either cell expansion medium or osteogenic medium was used, as shown in (Figure 5); one-way ANOVA and pairwise *t*-test with *p*-value < 0.05). 

### 4.3. ELISA Release Assay

**Figure 6:**
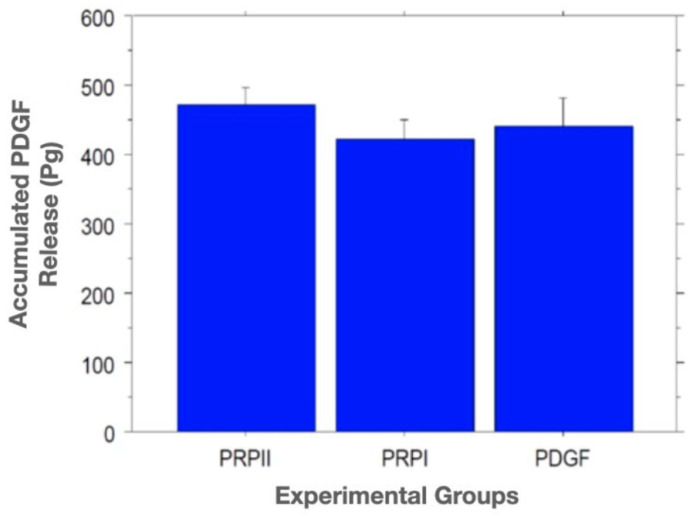
Graph showing no significant difference between the experimental groups for the accumulated release of PDGF from the gelatin–HPA among the groups. One-way ANOVA *p* value = 0.5228.

The standard curve fit was calculated using the four-parameter logistic regression curve. The concentrations of the PDGF in the collected supernatant at the various time points in the cumulative release amounts were calculated. The results showed that after 28 days, the average of the amount of cumulative released PDGF-BB from PRP lysate I, PRP lysate II, and the recombinant PDGF-BB groups were 423 ± 27 pg, 474 ± 27 pg, 441 ± 40 pg, respectively. Results also showed that 58%, 64%, and 98.8%, respectively, of the original PDGF-BB amount remained in the gels at 28 days for the PRP lysate preparations I, II, and recombinant PDGF-BB groups. One-way ANOVA showed no statistically significant difference in the cumulative release profile of PDGF-BB among the groups with *p* value = 0.5228 (Figure 6). Using Fisher’s PLSD statistical analysis to compare each pair of the groups also showed no significant differences in the release between groups.

## 5. Discussion

**Figure 7:**
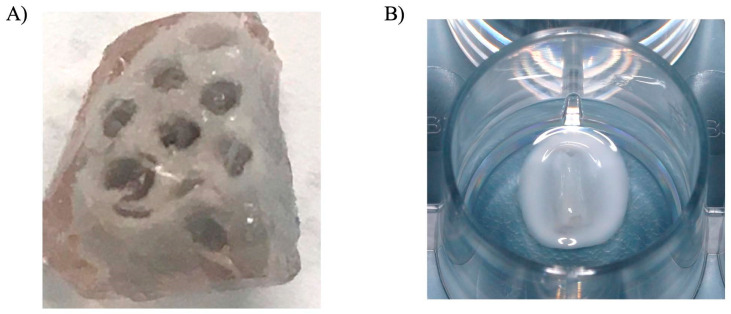
(**A**) Representative image showing the Calcium Phosphate porous block infused with the Gtn–HPA hydrogel (Pore size ~900 μm). (**B**) Image showing the Gtn–HPA infused block in a cell culture well for a migration assay surrounded by the MSC-seeded collagen ring.

The supposition of this study was that Gtn–HPA incorporating PRP lysate could be used to fill the pores and channels of porous blocks employed for bone reconstruction to replace the function of a fibrin clot. A preliminary experiment was carried out to infuse a CaP porous block with the Gtn–HPA hydrogel (Figure 7). With its tunable, covalent crosslinked density, which determines degradation rate and mechanical properties [24,28,29], Gtn–HPA can be formulated to serve as a longer-lasting scaffold than fibrin to support cell migration, differentiation, and synthesis of a new matrix. An attendant notable feature of Gtn–HPA is the wide range of modulus of elasticity that can be produced with this hydrogel, which may be of help in tuning osteogenic differentiation [28]. 

The results of ring/core migration assay of our first objective supported our hypothesis that Gtn–HPA accommodates MSC migration when an MSC chemoattractant is incorporated into the gel. PRP lysate-incorporating hydrogels attracted a significantly higher number of migrating cells compared with the control and PDGF-BB groups with a *p*-value = 0.0001*. While PDGF-BB has been shown to be a chemoattractant and mitogen for many cell types including MSCs, in our migration study, PDGF showed no significant difference in attracting the migration of MSCs into the gel compared with the control group. In contrast, PRP lysate incorporated in the gel attracted the migration of three-fold more cells into the gel. Our study showing that Gtn–HPA is permissive of MSCs’ migration is in line with the results of previous work by Lim et al. [32], in which the authors showed that adult neural stem cells (aNSCs) were able to migrate into Gtn–HPA gel in a pattern of chain migration. Although the same pattern was shown in our results, the PRP lysate-incorporating Gtn–HPA group showed a more extensive interconnected tuft-like migration into the core hydrogel (Figure 2).

**Figure 8:**
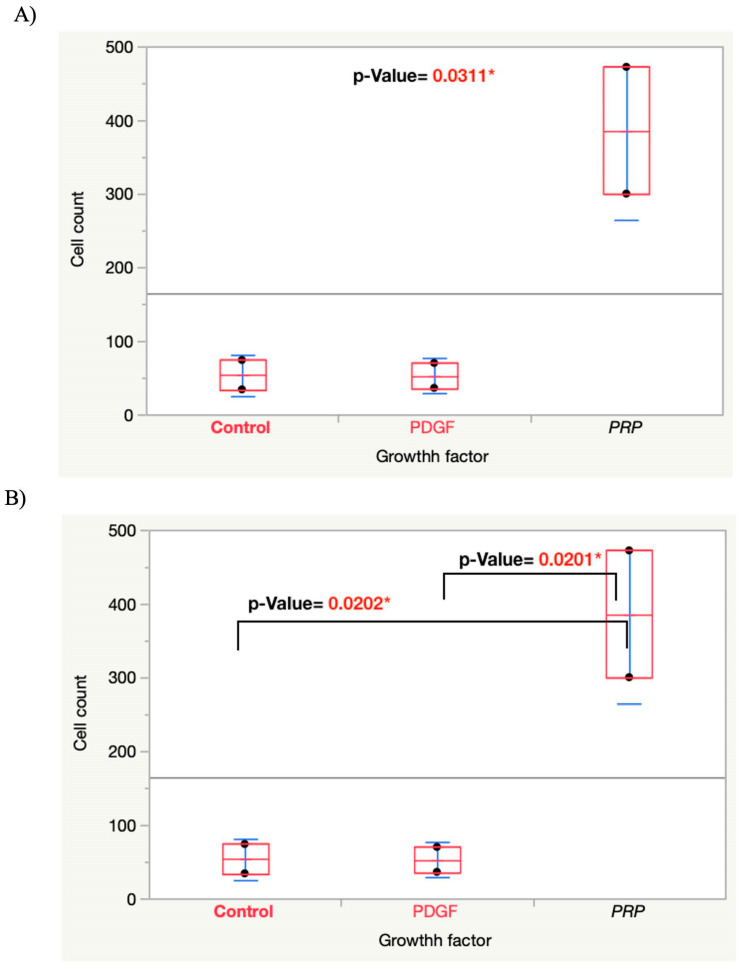
(**A**) Graph showing one-way ANOVA for the migration assay pilot study “cell count” by “Growth factor” (N = 2) among the groups. (**B**) Graph showing pairwise student t-test analysis between each pair of the groups.

Of note is that a pilot experiment using the migration assay was performed (N = 2 per experimental group) using a PDGF-BB concentration of 20 ng/mL instead of 50 ng/mL. In that preliminary study, the results were also similar to our reported final results (Figure 8). One explanation for the inability of PDGF-BB to chemoattract the MSCs into the gel may be related to the interaction of growth factors that may be inhibitory. In our study, during cell growth, medium was supplemented with FGF-2. This might have been one of the reasons why the PDGF-BB group did not show the usual significantly higher chemoattractant effect compared with the control group. The direct interaction between FGF-2 and PDGF-BB and their reciprocal inhibitory effect on the functions of different cell types has been reported in previous studies. Facchhiano et al. showed that FGF-b (FGF-2) inhibited the chemotactic and mitogenic effect of PDGF on rat aorta smooth muscle cells [35]. Another study by Faraone et al. strongly supported the evidence of cross-talk between the two growth factors and concluded that PDGF-BB has a reciprocal inhibitory effect on the mitogenic and other FGF-2-induced cell processes of human umbilical vein endothelial cells (HUVECs) by acting on the membrane level [36]. Other possibilities that can be proposed might be that the MSCs had a higher affinity to PRP lysate compared with PDGF or the mechanism by which the recombinant PDGF-BB acts on the MSCs to induce migration is different from the PRP lysate and might need more time to take effect.

In achieving our second objective, our results from the differentiation assay based on osteocalcin^+^ area percentage supported our hypothesis that the Gtn–HPA gel permitted MSCs within the gel to undergo osteogenic differentiation, driven by growth factors also incorporated in the gel. There was a statistically significant increase in osteocalcin^+^ area percentage for the PDGF-BB and PRP lysate-incorporating Gtn–HPA gels seeded with MSCs compared with both control groups, where either cell expansion medium or osteogenic medium was used (Figure 5); the one-factor ANOVA showed a significant difference among groups, with *p* = 0.024 (Figure 5). The pairwise t-test between each pair of the experimental groups also showed a significantly higher osteocalcin^+^ area percentage for the PDGF-BB or PRP lysate-incorporating Gtn–HPA compared with both control groups, where either cell expansion medium or osteogenic medium was used (Figure 5). Although the group supplemented with osteogenic medium showed both osterix^+^ and osteocalcin^+^ cells, the osterix fluorescence was more evident than osteocalcin. The significant difference between this group and the growth factor groups can be attributed to the longer time needed for the cells in this group to express osteocalcin, which is a late-stage osteogenic marker. However, this group expressed Osterix fluorescence, which indicates that the rat MSCs were able to differentiate into pre-osteoblasts in the Gtn–HPA.

Osteocalcin^+^ area percentage was chosen for the analysis, since it is a late osteogenic marker that is usually expressed at the time of calcium deposition and mineralization, which demonstrates the ability of the differentiated osteoblasts to form bone later. Our findings are in line with a study that investigated the effect of PRP on osteoblast differentiation in a three-dimensional scaffold. The authors concluded that PRP resulted in a significant increase in osteocalcin gene expression compared with control groups [37]. This study’s results support a previous study by our research group, Alshihri et al., where MSCs were able to proliferate, migrate, and differentiate in the injectable gelatin matrix in bony defects after shock wave stimulation [26,37].

The clinical benefits of using PRP remain unclear, in part due to the different preparation methods. In our study, PRP lysate showed a significant impact on MSC migration and osteogenic differentiation compared with the control groups. These findings are supported by many other previous studies [38,39,40]. Kaduko et al. studied the use of PRP combined with gelatin hydrogel granules and its effect on angiogenesis in murine subcutis. It was found that subcutaneous PRP and gelatin hydrogel granules significantly improved angiogenesis compared with control groups and concluded that this combination could be a promising potential treatment adjunct for ischemic disorders [38]. Another study by Matsui et al. [39] showed the enhanced angiogenic effect by multiple release of PRP contents and basic fibroblast growth factor from gelatin hydrogels. Therefore, we believe that PRP lysate is a cost-effective autologous source of growth factors that holds great promise for improving cellular functions and host regenerative ability [39]. 

Our growth factor release assay, in our third objective, showed that Gtn–HPA acted as an effective delivery vehicle for the sustained release of PDGF-BB for more than 28 days. An interesting finding was that the PDGF-BB release profile for the Gtn–HPA incorporating the two different PRP lysate preparations and for the recombinant PDGF-BB group showed no statistically significant difference among the groups, despite the large difference in the amount of PDGF-BB that was incorporated in the gels. This finding suggests that most of the PDGF-BB was bound in some way in the gel, and that only a freely mobile fraction was available to be released daily. In vivo, Gtn–HPA can serve as a stable depot for the growth factor, releasing it gradually as the gel undergoes enzymatic degradation by the action of the cells migrating into the gel. Future work will be required to determine the mechanism of action of this process. Our findings can further support the use of PRP lysate as a cost-effective autologous source of growth factors.

The current study was directed to investigate the benefit of injectable gels in filling the pores of porous blocks for treating bone defects. Other recent studies are investigating how certain particles, which might be employed in the future for producing those blocks, can influence osteogenesis. One such study [41], which incorporated gold, palladium, and superparamagnetic nanoparticles in a collagen scaffold, concluded that the nano-sized gold and palladium inhibited MSC proliferation, but superparamagnetic nanoparticles enhanced both osteoconductivity and osteoinductivity. These results could inform the types of particles employed for the fabrication of 3D printed blocks for bone reconstruction. 

## 6. Conclusions

Gtn–HPA hydrogel is permissive of MSC migration. PRP lysate incorporated in the hydrogel results in a significantly higher number of cells migrating into the Gtn–HPA compared with the blank gel and the recombinant PDGF-BB-containing hydrogel. Regarding the differentiation/osteogenesis assay, the PRP lysate and the PDGF-BB-incorporating gel result in a greater area% percentage of osteocalcin^+^ cells compared with the blank MSC-containing hydrogel. The release of the growth factors from PRP lysate, incorporated onto the gel, extends over the period of time necessary for bone reconstruction.

## Data Availability

The authors acknowledge that the data presented in this study must be deposited and made publicly available in an acceptable repository, prior to publication.
